# Biosynthesis and Roles of Salicylic Acid in Balancing Stress Response and Growth in Plants

**DOI:** 10.3390/ijms222111672

**Published:** 2021-10-28

**Authors:** Qinling Zhong, Hongliang Hu, Baofang Fan, Cheng Zhu, Zhixiang Chen

**Affiliations:** 1 College of Life Sciences, China Jiliang University, Hangzhou 310018, China; s20090710073@cjlu.edu.cn (Q.Z.); hlhu2020@cjlu.edu.cn (H.H.); 2 Purdue Center for Plant Biology, Department of Botany and Plant Pathology, Purdue University, West Lafayette, IN 47907-2054, USA; bfan@purdue.edu

**Keywords:** salicylic acid, plant immunity, salicylic acid biosynthesis, defense–growth tradeoff, auxin, defense response, PIN auxin transporters

## Abstract

Salicylic acid (SA) is an important plant hormone with a critical role in plant defense against pathogen infection. Despite extensive research over the past 30 year or so, SA biosynthesis and its complex roles in plant defense are still not fully understood. Even though earlier biochemical studies suggested that plants synthesize SA from cinnamate produced by phenylalanine ammonia lyase (PAL), genetic analysis has indicated that in Arabidopsis, the bulk of SA is synthesized from isochorismate (IC) produced by IC synthase (ICS). Recent studies have further established the enzymes responsible for the conversion of IC to SA in Arabidopsis. However, it remains unclear whether other plants also rely on the ICS pathway for SA biosynthesis. SA induces defense genes against biotrophic pathogens, but represses genes involved in growth for balancing defense and growth to a great extent through crosstalk with the growth-promoting plant hormone auxin. Important progress has been made recently in understanding how SA attenuates plant growth by regulating the biosynthesis, transport, and signaling of auxin. In this review, we summarize recent progress in the biosynthesis and the broad roles of SA in regulating plant growth during defense responses. Further understanding of SA production and its regulation of both defense and growth will be critical for developing better knowledge to improve the disease resistance and fitness of crops.

## 1. Introduction

Salicylic acid (SA) is produced by many prokaryotic and eukaryotic organisms including plants. In plants, SA has regulatory functions as a plant hormone [1]. More than 30 year ago, it was discovered that SA is the natural trigger of heat production in thermogenic plants by activating alternative respiration to volatilize putrid-smelling compounds to attract pollinating insects [2]. The best-established role of SA is as a defense signal molecule in plant immune responses [1]. More than 40 year ago, it was reported that the application of exogenous SA triggers immune-like responses in plants characterized by induced production of plant-pathogenesis-related (PR) proteins and induces disease resistance [3]. In resistant plants, pathogen infection often induces increased SA levels, not only locally in infected parts, but also in upper uninfected leaves that develop systemic acquired resistance (SAR) [4,5,6]. Plants compromised in SA accumulation either due to increased SA metabolism or reduced SA biosynthesis are often hypersusceptible to pathogen infection and unable to establish SAR [7,8].

With the established role of SA in plant immunity, there has been extensive research over the past 30 year to understand the molecular basis for SA-mediated immune responses. Specifically, unlike in some bacteria, plants synthesize SA through two pathways: from cinnamate produced by phenylalanine ammonia lyase (PAL) and from isochorismate (IC) produced by IC synthase (ICS) [9]. Recent progress has been made in identifying the critical components in the ICS pathway for SA biosynthesis in the model plant Arabidopsis [10,11]. However, it remains to be determined whether different plants rely on the ICS, PAL, or both pathways for SA biosynthesis. In addition, it has been increasingly recognized that SA-mediated immune responses are multilayered, involving not only the activation of specific defense mechanisms, but also the modulation of plant growth to balance plant defense and growth through crosstalk with other plant hormones such as auxin. Thus, SA is also a regulator of plant growth. In this work, we review recent progress in the research on SA biosynthesis and the emerging roles of SA in regulating plant growth with a particular focus on its crosstalk with auxin under stress conditions.

## 2. Biosynthesis of SA in Plants

**Figure 1:**
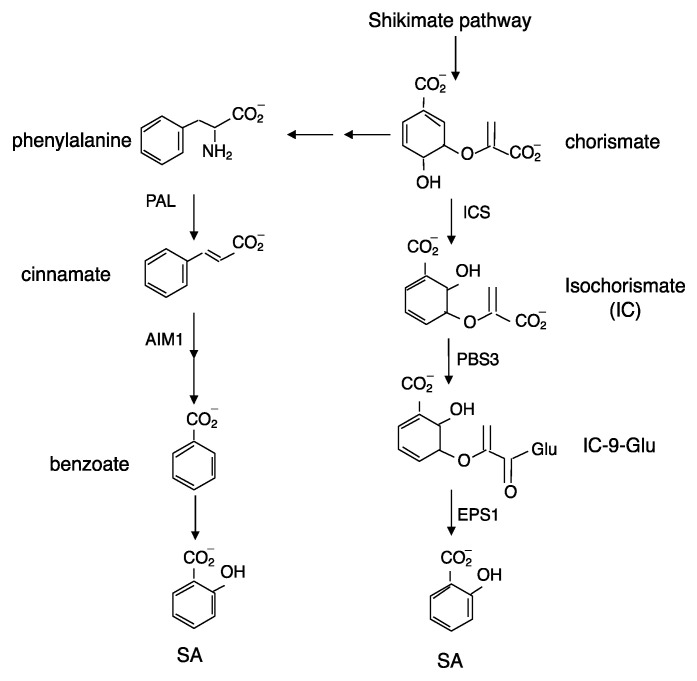
Pathways of SA biosynthesis in plants.

Both biochemical and genetic approaches have been used to understand the biosynthetic pathways of SA in plants. Biochemical studies using isotope feeding have suggested that SA is synthesized from cinnamate produced by PAL (Figure 1). Cinnamate can be converted to SA through o-coumarate or benzoate depending on whether hydroxylation of the aromatic ring occurs before or after the chain-shortening reactions, most likely through a β-oxidation process analogous to fatty acid β-oxidation [12]. In tobacco and rice, a benzoic acid 2-hydroxylase (BA2H) activity was detected and the tobacco BA2H activity was partially purified as a soluble 160 kDa protein that could be immunoprecipitated by antibodies against the soluble SU2 cytochrome P450 from *Streptomycin griseolus* [13]. However, there has been no further report on the purification of the BA2H protein or isolation of the corresponding gene(s). Therefore, even though it has been almost half a century since the biochemical evidence for the PAL pathway of SA biosynthesis was first reported [12], none of the enzymes required for the conversion of SA from cinnamate in the PAL pathway have been isolated from plants. However, in rice, a protein similar to Arabidopsis abnormal inflorescence meristem 1 (AIM1), which encodes a 3-hydroxyacyl-CoA dehydrogenase involved in β-oxidation, plays an important role in rice SA production [14]. A mutation of rice AIM1 led to reduced SA levels in roots and reduced root meristem activity [14]. A requirement of an enzyme involved in β-oxidation for SA biosynthesis strongly supports the PAL pathway in rice SA biosynthesis (Figure 1). To directly determine the role of the PAL pathway in SA biosynthesis, we previously generated two independent quadruple knockout mutants for the four *PAL* genes in Arabidopsis [15]. However, the two Arabidopsis *PAL* quadruple mutants still have about 25% of the wild-type basal SA levels and about 50% of induced SA levels after pathogen infection [15]. Thus, mutations of all the four *PAL* genes in Arabidopsis can affect, but not abolish SA production in Arabidopsis.

In bacteria, SA can be synthesized from chorismate through two reactions catalyzed by isochorismate synthase (ICS) and isochorismate pyruvate lyase (IPL) [16]. There are two *ICS* genes in Arabidopsis: *ICS1* (also known as *SID2*) and *ICS2* [7]. In the *ICS1* mutants, total SA levels were reduced by 90–95% when compared to those in wild-type plants after pathogen infection [7]. The residual levels of SA in pathogen-induced *ICS1* mutants might be synthesized by ICS2 or through another pathway. Indeed, when compared for UV-induced SA accumulation, the *ICS1* single mutant accumulated about 10%, but the *ICS1 ICS2* double mutant accumulated only about 4% of total SA compared to the wild-type [17]. Thus, the ICS pathway is responsible for roughly 95% of the SA synthesized in UV-treated Arabidopsis plants. Although the critical role of ICS indicates that the ICS pathway is responsible for the synthesis of a majority of SA in Arabidopsis (Figure 1), there is no gene encoding IPL in plants catalyzing the conversion of SA from isochorismate.

There are other Arabidopsis mutants with altered SA accumulation, and some of them have now been established to result from mutations of three genes encoding components in the ICS pathway. The first gene is *PBS3* (also known as GDG1 or WIN3), encoding an acyl-adenylate-/thioester-forming enzyme from the glycoside hydrolyase 3 (GH3) family [18,19,20]. As will be discussed later, some GH3 proteins can adenylate jasmonic acid (JA) and indoleacetic acid (IAA) and catalyze their conjugation to amino acids through amide bonds. The second gene is *EPS1*, which we first isolated with compromised resistance to the bacterial pathogen *Pseudomonas syringae* due to greatly reduced SA accumulation [21]. EPS1 is a member of the BAHD acyltransferase superfamily, which was named based on four plant enzymes (BEAT, AHCTs, HCBT, and DAT) characterized in this family that all catalyze CoA-dependent acylations [21]. Using a combined genetic and metabolomic approach, two groups have recently independently reported that PBS3 acts as an isochorismoyl-glutamate synthase that adenylates IC to catalyze its conjugation to glutamate to produce isochorismoyl-9-glutamate [10,11] (Figure 1). Isochorismoyl-9-glutamate is subsequently converted to SA either spontaneously or catalyzed by EPS1 [10,11] (Figure 1). Thus, unlike bacteria that convert IC to SA through a single step catalyzed by IPL, Arabidopsis has evolved a unique pathway of two steps of conversion of SA from IC catalyzed by PBS3 and EPS1 (Figure 1). While ICS is localized in chloroplasts, both PBS3 and EPS1 are in the cytosol. Therefore, IC generated by ICS in chloroplasts requires transport to the cytosol, which is carried out by the product of another gene important for SA biosynthesis, *EDS5* [22,23]. EDS5 is a chloroplast envelop-localized MATE (multidrug and toxin extrusion) transporter family protein that functions as an IC transporter [22,23].

While the critical role of the ICS pathways and the long-sought-after enzymes that catalyze its last two steps in SA biosynthesis have now been fully established in Arabidopsis, it remains to be determined whether other plants also primarily use the ICS pathway for SA biosynthesis. Unlike in Arabidopsis, infection by the hemibiotrophic pathogens *P. syringae* and *Phytophthora sojae* in soybean leads to reduced *ICS* gene expression [24]. On the other hand, pathogen-induced SA biosynthesis is associated with reduced phenylalanine content [24]. Silencing of five PAL isoforms or two ICS isoforms is equally effective in suppressing SA biosynthesis and compromising disease resistance [24]. These results indicate that the PAL and ICS pathways are equally important for pathogen-induced SA biosynthesis in soybean. In rice shoots, SA levels are several-hundred-fold higher than those in Arabidopsis and tobacco even under normal growth conditions without pathogen infection [25]. Unlike Arabidopsis with two *ICS* genes, rice contains a single *ICS* gene. Despite the extremely high SA levels in rice shoots, the rice ICS has a very low level of enzymatic activity when compared to the Arabidopsis homolog [26]. In addition, a phylogenetic analysis has shown that plant GH3 proteins can be classified into three groups [27]. PBS3 is a member of group III GH3 proteins that are exclusively found in dicot plants, but not in monocot plants such as rice [28]. EPS1, on the other hand, belongs to a unique subfamily of BAHD acyltransferases found only in the Brassicaceae family of plants and contains an unusual active site amino acid change from BAHD acyltransferases [21]. These findings would argue against the operation of the same ICS pathway in rice SA biosynthesis. On the other hand, as described earlier, a mutation in the rice *AIM1* gene led to reduced SA levels in roots [14]. A requirement of an enzyme associated with β-oxidation for SA biosynthesis strongly suggests a critical role of the PAL pathway in rice SA biosynthesis. Therefore, despite research over the past half a century and the recent breakthrough in establishing the ICS pathway in Arabidopsis, important questions remain to be addressed about the pathways of SA biosynthesis in other plants.

## 3. Defense Crosstalk with Auxin in Plants

Activation of plant defense protects plants from pathogen infection generally at the expense of plant growth, probably due to competition for the limited amount of resources [29,30]. In order to both survive and grow, plants have evolved complex mechanisms to balance growth and defense. Many studies have revealed that defense crosstalk with auxin plays an important role in the regulation of the growth–defense tradeoff [31]. For example, many pathogens including *P. syringae* and *Agrobacterium tumefaciens* can either directly produce auxin or manipulate plant auxin synthesis and signaling to promote plant susceptibility [32,33,34,35,36]. The application of exogenous auxin to plants prior to inoculation of virulent strains of *P. syringae* can also lead to increased plant susceptibility to the bacterial pathogen [37]. On the other hand, the activation of plant defense is often associated with suppressed auxin signaling and response. During flg22-triggered immunity, both the transcript and protein levels of the auxin receptors are reduced, leading to increased stability and accumulation of AUX/IAA repressor proteins and repression of auxin-responsive genes [38]. This suppression of auxin signaling and response is in part due to the induced expression of the microRNA miR393, which directly targets the cleavage of the transcripts for auxin receptors TIR1, AFB1, and AFB3 [38]. Overexpression of miR393 increases plant resistance, while overexpression of AFB1 enhances susceptibility to virulent pathogens [38]. These findings indicate that suppression of auxin signaling is important for plant immunity.

Several studies have also revealed that during the activation of defense responses, increased SA production and signaling are associated with a concomitant reduction in auxin biosynthesis, transport, and signaling, thereby coordinating defense and growth. For example, in cassava, heat shock protein MeHSP90.9 regulates immune response through fine-tuning the antagonistic interaction between SA and auxin biosynthesis [39]. Cassava bacterial blight (CBB) induces the expression of MeHsf8, which activates MeHSP90.9 expression and immune response [39]. MeHSP90.9 interacts with and activates the MeSRS1 and MeWRKY20 transcription factors to promote the expression of the SA biosynthetic gene avrPphBSusceptible3 (MePBS3) and the tryptophan metabolic gene N-acetylserotonin O-methyltransferase 2 (MeASMT2) [39]. Induced expression of MePBS3 activates SA biosynthesis, but increased MeASMT2 expression inhibits tryptophan-derived auxin biosynthesis, highlighting the dual regulation of SA and auxin biosynthesis by MeHSP90.9 during the immune response. In Arabidopsis, the protein kinase CK2 also regulates both the SA and auxin pathways [40,41,42]. CK2 modulates SA homeostasis, and the functional interplay between CK2 and SA also regulates the expression of PIN-formed (PIN) genes, which encode auxin efflux transporters [42]. CK2 also plays an important role in the transcriptional regulation of PINOID (PID), an AGC protein kinase involved in the regulation of the apical/basal localization of auxin-efflux transporters [40]. Furthermore, CK2 activity is required for proteosome-dependent degradation of AXR3, a member of the AUX/IAA family of auxin transcriptional repressors [40]. These results indicate a role for CK2 in the coordination of the antagonistic regulation between auxin- and SA-related signaling and responses.

In addition to the coordinated regulation of SA and auxin signaling during the activation of plant immune responses, there is a substantial number of reports on the direct effects of SA on auxin biosynthesis, distribution, and signaling. These studies reveal the dual activities of SA in both the induction of defense mechanisms and the suppression of the growth-promoting activity of auxin, thereby establishing the direct roles of SA in balancing defense and growth in plants. The extensive research on SA-mediated defense responses has resulted in the identification of components such as nonexpresser of PR gene (NPR) proteins important for SA signaling, which has been reviewed recently [43]. The studies on the role of SA in the defense–growth tradeoff have also identified important new components in SA signaling that are distinct from those involved in SA-mediated defense signaling, thereby broadening our understanding of the complex networks of SA-mediated signal transduction. In the following sections, we summarize these recent studies on the regulation of auxin biosynthesis, metabolism, transport, and signaling by SA in plants. We focused our discussion of the progress in Arabidopsis, on which a vast majority of the studies with important new findings on these topics have been conducted.

## 4. SA Regulation of Auxin Biosynthesis and Metabolism

**Figure 2:**
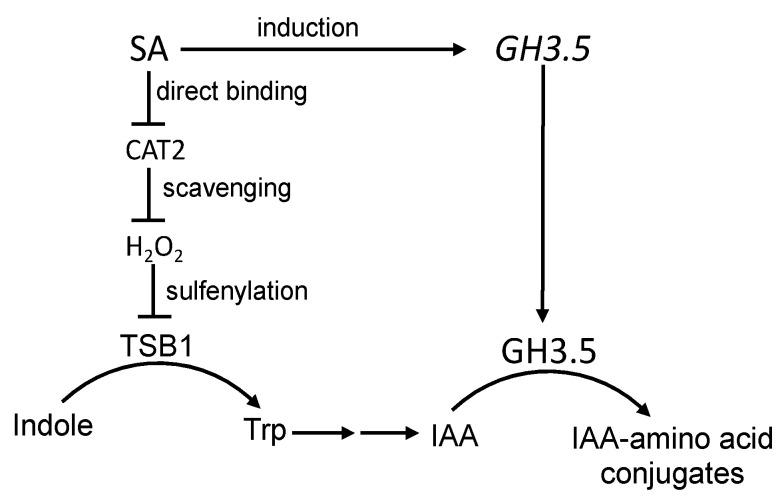
Regulation of auxin biosynthesis and metabolism by SA. SA directly binds to and inhibits CAT2 to increase H_2_O_2_ levels, which promotes sulfenylation of an IAA biosynthetic enzyme, TSB1, to inhibit its activity, thereby reducing IAA production. SA also induces the expression of *GH3.5*, which encodes an acyl acid amido synthetase that conjugates amino acids to IAA, causing its inactivation or degradation.

It has been reported that SA represses the expression of auxin-related genes, but has no significant effect on free auxin levels 24 and 48 h after SA treatment [44]. However, free auxin levels were reduced in several SA overaccumulating mutants such as *cpr6* and *snc1*, which exhibit reduced apical dominance and stunted growth, typically caused by auxin deficiency [44]. These observations indicate that even though exogenous application of SA does not immediately affect free auxin levels, chronic SA overproduction can influence auxin homeostasis. A more recent study discovered that biotrophic pathogen-induced SA can reduce the biosynthesis of both auxin and JA through catalase 2 (CAT2) in Arabidopsis [45] (Figure 2). Catalases from tobacco were the first plant proteins found to bind SA and those biologically active SA analogs capable of activating plant defense responses [46]. SA can bind to SA-binding plant catalases and inhibits their activity to elevate the cellular H_2_O_2_ levels [46]. Further analysis has revealed that SA inhibits catalases by acting as an electron-donating substrate that donates a single electron to catalases to trap the enzyme in an inactive redox state, and in doing so, SA is also converted into SA radicals [47]. Both the elevated H_2_O_2_ levels as a result of catalase inhibition and the generation of SA radicals may contribute to the activation of SA-mediated defense responses.

In their published study, Yuan and coworkers provided extensive evidence that Arabidopsis CAT2 functions as an SA receptor that mediates the SA-mediated inhibition of both auxin and JA biosynthesis [45]. First, in wild-type plants, pathogen infection leads to the inhibition of catalase activity and increased H_2_O_2_ accumulation [45]. However, in the SA-deficient *sid2* mutant, pathogen-induced catalase inhibition is alleviated and the accumulation of H_2_O_2_ is prevented [45]. Second, the phenotypes of the enhanced disease susceptibility and compromised inhibition of auxin and JA biosynthesis of the *sid2* single mutant are partially rescued by the introduction of the *cat2* mutation [45]. This result demonstrates that inhibition of CAT2 activity is a critical mechanism by which SA induces disease resistance. Third, increased H_2_O_2_ accumulation from SA inhibition of CAT2 increases sulfenylation of the IAA biosynthesis enzyme tryptophan synthetase b subunit 1 (TSB1), which leads to the inhibition of the TSB1 enzymatic activity and reduced IAA production during SA-mediated resistance to biotrophic pathogens [45] (Figure 2). Enzymatically active CAT2 also physically interacts with the JA biosynthesis enzymes acyl CoA oxidases 2 and 3 (ACX2/3) to stimulate their activities, presumably through actively removing H_2_O_2_ generated from the ACX2-/3-calayzed reaction [45]. SA binding of CAT2 inhibits its activity, thereby suppressing the activity of CAT2 to stimulate ACX2/3 and promote JA biosynthesis [45].

The levels of plant hormones are controlled not only through their biosynthesis and degradation, but also by their conjugation to different molecules such as amino acids. In Arabidopsis, GH3.5/WES1 acyl acid amido synthetase conjugates aspartate to both IAA and SA [48,49,50]. Attachment of aspartate and glutamate to IAA, for example, can lead to degradation of auxin [51]. By contrast, salicyloyl-aspartate synthesized by GH3.5 is a potential activator of plant immunity in Arabidopsis [52]. Expression of *GH3.5* is induced by both SA and IAA [48,50] (Figure 2). Two gain-of-function mutants for GH3.5 (*wes1-D* and *gh3.5-1D*) identified from activation tagging displayed low auxin phenotypes including reduced growth and altered leaf shape, but increased resistance to both biotic and abiotic stresses [48,50]. By contrast, T-DNA insertion mutants for GH3.5 displayed reduced stress resistance including compromised SAR associated with diminished *PR* gene expression in systemic leaves [48,50]. The results from biochemical, molecular, and genetic analysis of GH3.5 are consistent with its role as a positive regulator of plant immunity through modulating SA-IAA crosstalk. Gain-of-function mutants and overexpression lines for GH3.5 also accumulate more SA, and the reasons for this phenotype are not fully understood. Biochemical and structural analysis of GH3.5 has shown that this protein can conjugate SA, but is more efficient in conjugating benzoic acid [49]. It has been proposed that the conversion of benzoic acid to its aspartate conjugate may contribute to SA biosynthesis [49]. Given the recent finding that another GH3 protein, PBS3, functions as an IC-glutamate synthase that adenylates IC to catalyze its conjugation to glutamate to produce IC-9-glutamate in the ICS pathway of SA biosynthesis, it has been suggested that there is a pathogen-induced SID2-dependent, but PBS3-independent SA biosynthetic pathway in Arabidopsis [20], likely due to the existence of other members of the GH3 family that also function in the ICS pathway of SA biosynthesis [10]. Given the positive role of GH3.5 in SA production, it is tempting to speculate that GH3.5 may contain a promiscuous activity of IC-glutamate synthetase that functions in SA biosynthesis. Interestingly, while PBS3 is inhibited by SA [53], GH3.5 is induced by SA [48,50] and, therefore, may play a positive role in SA production even under high SA levels in pathogen-infected plants.

## 5. SA Regulation of Auxin Transport

**Figure 3:**
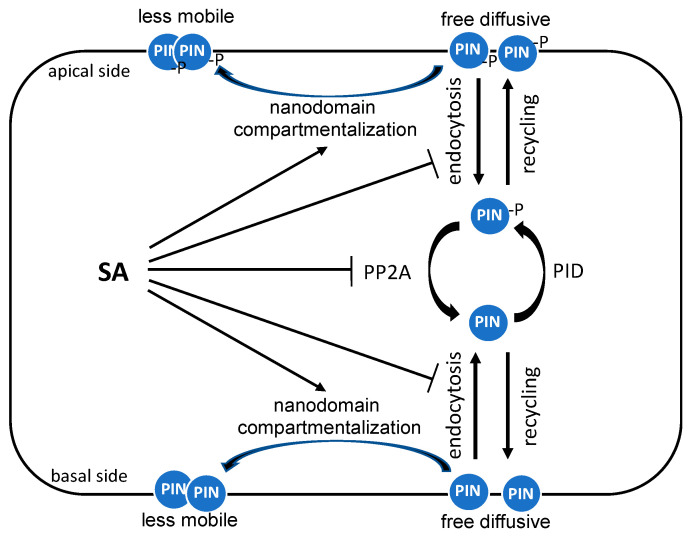
Regulation of the auxin polar distribution by SA. The auxin polar distribution, which is important for auxin-mediated growth and development, is largely mediated by the polar distribution of PIN auxin efflux transporter proteins through regulated endocytic recycling. SA disrupts endocytic recycling of PIN proteins by inhibiting their endocytosis, affecting their phosphorylation through inhibition of PP2A and inducing their hyperclustering in the plasma membrane.

Most of the research on the roles of SA in plant growth, development, and stress responses has been focused on plant shoots. However, SA also affect root growth and the response to biotic and abiotic conditions. Particularly relevant to its role as a defense phytohormone, SA modulates the colonization of the root microbiome by specific bacterial families [54]. In addition, several groups have reported that SA attenuates root growth, gravitropic response, and lateral root organogenesis through crosstalk with auxin transport and distribution. These effects of SA on root growth and development involve PIN auxin transporters. A dynamic control of cellular PIN polarity affects the directionality of auxin fluxes and modulates auxin-regulated growth and developmental processes [55,56,57]. The polar distribution of PIN proteins is primarily established by clathrin-mediated endocytosis and their recycling to the plasma membrane as the initial secretion of newly synthesized PIN proteins is not polar [58] (Figure 3). Importantly, SA has been found to inhibit clathrin-mediated endocytosis of plasma membrane proteins including PIN proteins [59] (Figure 3). The inhibitory effect of SA on clathrin-mediated endocytosis is not NPR1-dependent, indicating that SA’s interference with clathrin-mediated protein trafficking is independent of the well-established SA signaling pathway [59]. In plants, clathrin-mediated endocytosis also requires accessory adaptor proteins, adaptor protein 2 (AP-2) and the TPLATE complex (TPC). SA reduces the membrane association of clathrin and AP-2, but not that of the TPC, whereas auxin, which also inhibits clathrin-mediated endocytosis, solely affects clathrin membrane association [60]. Therefore, SA interferes with the association of clathrin and its adaptor proteins to the plasma membrane to inhibit clathrin-mediated endocytosis. Consistent with the inhibitory mechanism, clathrin-deficient mutants are less sensitive to SA on the auxin distribution and root gravitropic response [59]. Interestingly, SA does not inhibit the ligand-induced endocytosis of the flagellin sensing 2 (FLS2) receptor during plant immune responses [59].

More recent studies have revealed additional mechanisms by which SA affects auxin transport through regulation of the polar plasma membrane distribution of PIN proteins. For example, SA can regulate root growth and development by altering the phosphorylation of PIN proteins to affect auxin transport (Figure 3). Reversible phosphorylation of PIN proteins plays an important role in regulating their polarity, subcellular dynamics, and activity. PIN proteins can be phosphorylated by several kinases, including PID (PINOID)/wavy root growths (WAGs), D6PK/D6PKLs, and protein kinase associated with BRX (PAX) and dephosphorylated by multiple phosphatases, including protein phosphatase 2A (PP2A), PP1, and PP6 [41,61,62,63]. SA directly binds to A subunits of PP2A and inhibits the activity of this complex [64] (Figure 3). The PIN2 auxin transporter is a PP2A target and is consequently hyperphosphorylated in response to SA, leading to the changed activity of the auxin efflux transporter and inhibition of auxin transport and auxin-mediated root development, including growth, gravitropic response, and lateral root organogenesis [64] (Figure 3). Again, SA’s action on PP2A, the polar distribution of root auxin, and PIN proteins, and ultimately root growth, are independent of the canonical NPR receptors [64].

Another mechanism by which SA can alter auxin-mediated root growth is through regulating PIN protein hyperclustering in the plasma membrane [65] (Figure 3). There are two populations of PIN proteins, a less mobile form and a free diffusive pool [66] (Figure 3). The heterogeneous distribution of PIN is modulated by the clustering of dynamic membrane subcompartments called nanodomains, which are enriched in specific lipids and protein components. Nanodomains are actively involved in plant signaling by concentrating their signaling molecules into the lipid order phase on the plasma membrane [67]. Significantly, pathogen infection induces the assembly of nanodomains, leading to increased intermolecular and intramolecular interactions of membrane-associated signaling proteins for defense signaling [68]. SA also triggers the compartmentalization of lipid raft nanodomains and increases the lipid order phase of the plasma membrane through a modulation of the lipid raft regulatory protein, remorin (Figure 3). Specifically, SA induces remorin clustering and membrane nanodomain compartmentalization to regulate plasmodesmata closure to impede virus spreading [69]. Very recently, it has been revealed that SA can also regulate auxin signaling by constraining the plasma membrane dynamics of the PIN2 auxin efflux transporter in Arabidopsis roots [65]. SA causes increased constraining of the lateral diffusion of PIN2 proteins, which is associated with increased accumulation of PIN2 proteins into hyperclusters in a manner dependent on REM1.2-mediated nanodomain compartmentalization [65]. This SA-induced membrane nanodomain compartmentalization of PIN2 also inhibits clathrin-mediated endocytosis [65]. As a result, SA-induced heterogeneous surface condensation causes the disruption of asymmetric auxin distribution, root growth, and gravitropic response [65]. These results demonstrate another defense–growth tradeoff mechanism by which SA interferes with auxin transport by condensing PIN auxin efflux transporter proteins into heterogeneous compartments.

## 6. SA Regulation of Auxin Signaling and Response

Both endogenously produced and exogenously applied SA triggers plant immune-like responses by activating the reprogramming of large-scale gene expression. NPR1 is required for SA-induced defense responses [70]. Recombinant NPR1 binds SA [71,72,73], and this SA-binding activity is required for the activation of SA-responsive defense genes by NPR1 [71]. Two close NPR1 paralogs, NPR3 and NPR4, have a similar domain structure as NPR1. However, unlike NPR1, NPR3 and NPR4 function as negative regulators of immunity by acting as transcriptional repressors of SA-responsive defense genes [71,74]. NPR3 binds SA with a similar affinity as NPR1, while NPR4 binds SA with an affinity five-times higher than NPR1 [71,75]. The presence of two types of SA receptors with opposite functions would allow for tight regulation of SA-induced defense responses at different SA levels [43]. At low SA levels, NPR3 and NPR4 repress unnecessary and potentially harmful defense gene activation. However, at high SA levels, the transcriptional repressor activities of NPR3 and NPR4 are inhibited to allow for the release of the repression of the SA-responsive target genes. SA causes increased expression of a large number of plant genes including *PR* genes with diverse roles in defense signaling and responses. Importantly, several studies have reported that SA can also repress the expression of genes involved in auxin signaling and response.

**Figure 4:**
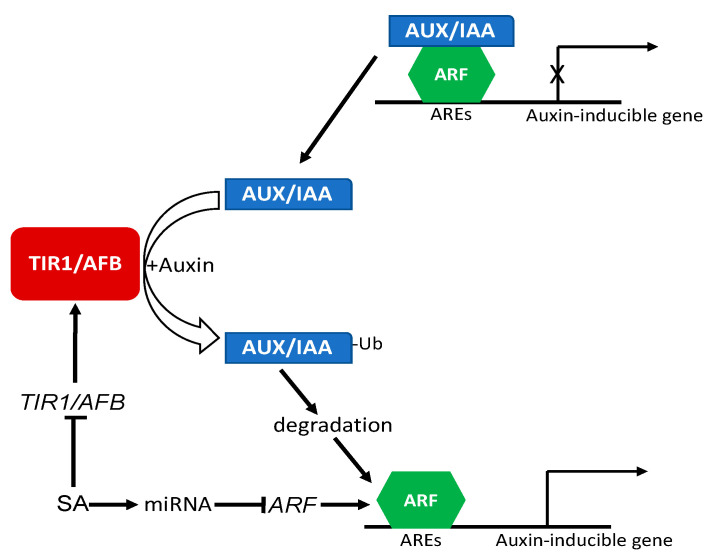
Regulation of auxin signaling and response by SA. In the absence of auxin, auxin-inducible genes are repressed by AUX/IAA repressors through interaction with ARF transcription factors. When auxin levels increase, auxin binds to the TIR1/AFB auxin receptor complexes to promote their binding of AUX/IAA repressors and targets their ubiquitination and degradation. Degradation of AUX/IAA repressors leads to derepressing ARF-dependent transcription of auxin-regulated genes. SA represses the expression of auxin TIR1/AFB auxin receptor genes. SA is also dependent on targeting of ARF genes by microRNAs such as miR167.

Auxin signaling and responses are mediated by auxin receptors and auxin-regulated transcription factors. At low auxin levels, auxin response genes are actively repressed by the AUX/IAA family of transcriptional repressor proteins, which form complexes with the auxin responsive factors (ARF) family of transcription factors [76,77,78] (Figure 4). When auxin levels increase in the cells, auxin directly bind to the F-box auxin receptor proteins, transport inhibitor resistant 1 (TIR1) and auxin signaling F-box (AFB), which are substrate-recognition components of an SKP–Cullin–F-box (SCF) E3 ubiquitin ligase complex, SCFTIR1/AFB [76]. Auxin binding to its TIR1/AFB receptor proteins promotes SCFTIR1/AFB binding to AUX/IAA repressor proteins, thereby targeting their ubiquitination and degradation by the 26S proteasome to derepress ARF-dependent transcription of auxin-regulated genes [76] (Figure 4). Using the Affymetrix ATG1 Arabidopsis Gene-Chip, Wang and coworkers reported global repression of auxin-related genes by SA [44]. Among the SA-repressed auxin-related genes are those involved in auxin signal transduction such as *AUX1* and *PIN7*, which encode an auxin importer and exporter, respectively. More importantly, they also include genes for the TIR1 and AFB1 auxin receptors, as well as the auxin-inducible SAUR and Aux/IAA family genes [44]. These results indicate that SA negatively regulates auxin signaling by repressing the expression of auxin receptor genes (Figure 4). On the other hand, the expression of genes encoding auxin-conjugating enzymes was upregulated by SA, implying that SA might lower the free auxin levels [44]. A majority of these auxin-related genes were also repressed after the induction of SAR [44], indicating that downregulation of auxin-related genes might be a part of the SA-induced defense response. Indeed, an increase in auxin levels or auxin sensitivity is known to promote plant susceptibility to pathogen infection, while reducing auxin sensitivity (e.g., in the *axr2-1* mutant expressing a nondegradable AXR2/IAA7 repressor protein) increases plant disease resistance [44]. Furthermore, while transgenic Arabidopsis plants expressing the bacterial NahG SA hydroxylase are highly susceptible to pathogen infection due to SA deficiency, the introduction of the auxin-insensitive *ax2-1* mutation into the NahG lines drastically increases the disease resistance of SA-deficient plants [44]. These results indicate that inhibiting auxin sensitivity is a crucial component of the SA-mediated defense response.

In a more recent study, it was found that SA is required for the regulation of the target genes ARF6 and ARF8 by the microRNA miR167 for its activation of defense responses and disease resistance in Arabidopsis [79] (Figure 4). The microRNA miR167 regulates diverse processes including flower development, root development, and response to osmotic stress by controlling the expression of target genes ARF6, ARF8, and IAA-Ala resistant 3 [79,80,81,82,83]. miR167 also regulates defense against pathogens through ARF6 and ARF8. miR167 is differentially expressed in response to the bacterial pathogen *P. syringae*, and overexpression of miR167 confers increased resistance to the bacterial pathogen [79]. This resistance results from suppression of auxin responses associated with reduced expression of ARF6 and ARF8 and is also associated with altered stomatal behavior [79]. Importantly, these effects of miR167 overexpression on disease resistance, repression of ARF6 and ARF8, and stomatal closure are SA-dependent [79] (Figure 4). Therefore, SA also plays an important role in the repression of transcription factors such as ARF6 and ARF8 to suppress auxin response.

## 7. Summary and Prospect

Even though the critical roles of SA in plant immunity have been long recognized and established, important questions about the SA biosynthetic pathways in plants have not been fully understood. Biochemical, molecular, and genetic evidence indicates that both the PAL and ICS pathways contribute to SA biosynthesis. In Arabidopsis, the ICS pathway is responsible for the vast majority of SA produced under stress conditions, and the recent identification of the enzymes catalyzing the last two steps of the ICS pathway will facilitate future research on the dynamic regulation of SA biosynthesis in the model plant and its close relatives. However, it remains to be determined whether other plants also rely on the ICS pathway for SA biosynthesis. In fact, studies on rice and soybean have provided evidence in support of a critical role of the PAL pathway in SA biosynthesis in these plants. The evolutionary implications for the presence of more than one SA biosynthetic pathway are unclear, but will surely raise important questions on how these different pathways are coordinated and whether there are differences in the functionality and signaling mechanisms of SA synthesized from different pathways.

While it has been long recognized that SA can activate immune responses in plants to enhance disease resistance, it is becoming increasingly clear that SA has a broader role in plant immunity. Important progress has been made in the discovery of SA crosstalk with auxin, particularly in balancing defense and growth. Recent studies have discovered that SA suppresses the growth-promoting activity of auxin through downregulation of production, transport, and signaling. These studies have also identified important new components in SA signaling that are distinct from the well-established NPR receptors required for SA-mediated defense signaling. Over the past 30 year or so, a large number of SA-binding proteins have been identified in plants [84,85]. Some of these SA-binding proteins have now been established to play critical roles in SA signaling in plants. There are other growth-promoting plant hormones that have been implicated in the plant defense–growth tradeoff, and it would be of great interest to determine possible SA crosstalk with the plant hormones other than auxin in balancing growth and defense. These studies will further broaden our understanding of the complex networks of both SA-mediated signal transduction and the global regulation of plant growth, development, and stress responses.

## Data Availability

Not applicable.
